# Combination Therapy of Sophoraflavanone B against MRSA: *In Vitro* Synergy Testing

**DOI:** 10.1155/2013/823794

**Published:** 2013-11-10

**Authors:** Su-Hyun Mun, Ok-Hwa Kang, Dae-Ki Joung, Sung-Bae Kim, Yun-Soo Seo, Jang-Gi Choi, Young-Seob Lee, Seon-Woo Cha, Young-Sup Ahn, Sin-Hee Han, Dong-Yeul Kwon

**Affiliations:** ^1^BK21 Plus Team, Professional Graduate School of Oriental Medicine, Wonkwang University, 344-2 Sinyong-dong, Iksan, Chonbuk 570-749, Republic of Korea; ^2^Department of Oriental Pharmacy, College of Pharmacy and Wonkwang-Oriental Medicines Research Institute, Wonkang University, 344-2 Sinyong-dong, Iksan, Chonbuk 570-749, Republic of Korea; ^3^Department of Herbal Crop Research, National Institute of Horticultural & Herbal Science, RDA, Eumsung, Chungbuk 369-873, Republic of Korea

## Abstract

Sophoraflavanone B (SPF-B), a known prenylated flavonoid, was isolated from the roots of *Desmodium caudatum*. The aim of this study was to determine the antimicrobial synergism of SPF-B combined with antibiotics against methicillin-resistant *Staphylococcus aureus* (MRSA). MRSA, a multidrug-resistant pathogen, causes both hospital- and community-acquired infections worldwide. The antimicrobial activity of SPF-B was assessed by the broth microdilution method, checkerboard dilution test, and time-kill curve assay. The MIC of SPF-B for 7 strains of *S. aureus* ranges from 15.6 to 31.25 **μ**g/mL determined. In the checkerboard method, the combinations of SPF-B with antibiotics had a synergistic effect; SPF-B markedly reduced the MICs of the **β**-lactam antibiotics: ampicillin (AMP) and oxacillin (OXI); aminoglycosides gentamicin (GET); quinolones ciprofloxacin (CIP) and norfloxacin (NOR) against MRSA. The time-kill curves assay showed that a combined SPF-B and selected antibiotics treatment reduced the bacterial counts below the lowest detectable limit after 24 h. These data suggest that the antibacterial activity of SPF-B against MRSA can be effectively increased through its combination with three groups of antibiotics (**β**-lactams, aminoglycosides, and quinolones). Our research can be a valuable and significant source for the development of a new antibacterial drug with low MRSA resistance.

## 1. Introduction

**Figure 1:**
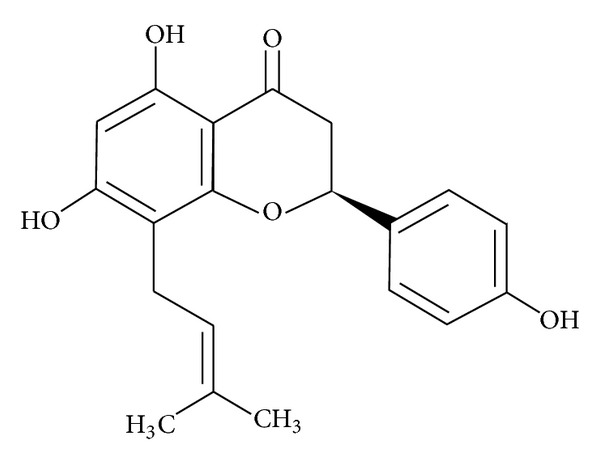
Chemical structure of sophoraflavanone B.

Sophoraflavanone B (SPF-B) is a prenylated flavanone discovered in 1978 by Komatsu et al. (Figure 1) [1]. SPF-B is known as a potential drug in menopausal hormone therapy, a strong phytoestrogen, and a compound with antibreast cancer activity. It is highly estrogenic, with great anti-androgenic potency [2–5]. Research into the antibacterial activity of many of the prenylated flavonoids, including SPF-B, has demonstrated a weak inhibition of MRSA. The anti-MRSA activity of sophoraflavanone B, isolated from the roots of *Desmodium caudatum*, was firstly reported by Sasaki et al. [6].

Methicillin-resistant *Staphylococcus aureus* (MRSA) was first reported in 1961, shortly after the introduction of methicillin into clinical use. Shortly afterward, MRSA was shown also to be resistant to many penicillins and cephems, as well as to methicillin. In recent years, MRSA accounts for nearly 70% of *Staphylococcus aureus* clinical cases, and the pathogen is the main cause of community-acquired and healthcare-associated infections [7]. MRSA is resistant to most *β*-lactam antibiotics (penicillins, cephalosporins, and carbapenems); *Staphylococcus aureus* becomes resistant by generating penicillin-binding proteins (PBP, PBP2′), which exhibit extremely low affinities for the *β*-lactam antibiotics *mecA *gene or *mec *[8]. The emergence of multidrug resistance in Gram-positive bacteria such as MRSA has demonstrated the overprescription of antibiotics, a practice that leads to strains that continuously develop new mechanisms of resistance. The known mechanisms of bacterial resistance to antibiotics include the inactivation of antibiotics by enzymes, a change in the target site, a change of membrane permeability, and antibiotic efflux to the outside of cells [9, 10]. However, the lethal target for antibiotics is not yet clear. For that reason, further studies are essential to the effort to minimize bacterial resistance to conventional drugs.

In this study, the increase in the antibacterial effect of SPF-B when combined with antibiotics on MRSA was investigated.

## 2. Materials and Methods

### 2.1. Materials and Reagents

Mueller-Hinton broth (MHB) was purchased from Difco (Baltimore, MD, USA) and ampicillin (AMP), oxacillin (OXI), gentamicin (GET), ciprofloxacin (CIP), norfloxacin (NOR), and sophoraflavanone B (≥95.0% SPF-B) were from Sigma-Aldrich Co. (St. Louis, USA).

### 2.2. Bacterial Strains and Growth Conditions

Among the 7 strains of *S. aureus* used in this study, 5 clinical MRSA isolates were obtained from 5 different patients at Wonkwang University Hospital. The remaining 2 strains were commercially purchased *S. aureus* ATCC 33591 (methicillin-resistant strain) and *S. aureus* ATCC 25923 (methicillin-susceptible strain, MSSA) (American Type Culture Collection, Manassas, VA). All bacteria were stored in 30% glycerol and frozen at −70°C until use. The bacterial strains were suspended in Mueller-Hinton broth (MHB) and incubated at 37°C for 24 hr.

### 2.3. Antimicrobial Resistance Testing

Detection of the *mecA* gene in MRSA strains was performed by PCR (polymerase chain reaction) amplification (Table 1). Prior to DNA extraction, bacteria stock cultures were subcultured twice on to MHA plates. For rapid extraction, one to five bacterial colonies were suspended in 300 *μ*L of cell lysis buffer and heated at 100°C for 20 min. After centrifugation at 12,000 rpm for 10 min, 2 *μ*L of the supernatant was used for the DNA extraction. PCR reactions were performed using a MRSA Primer Mix Kit (Genotek, Daejeon, Republic of Korea). The PCR amplification consisted of 30 cycles (94°C, 60 s; 55°C, 60 s; 72°C, 60 s). The final PCR products were separated on a 2% agarose gel.

### 2.4. Susceptibility Testing

The MIC determinations were performed using the broth microdilution method described by the Clinical and Laboratory Standard Institute guidelines [11]. Serial 2-fold dilutions of SPF-B in MHB were prepared in sterile 96-well microplates and microtubes. The MRSA inocula were adjusted to the 0.5 McFarland standard (approximately 1.5 × 10^8^ colony-forming units (CFU)/mL) in MHB. The final inocula were adjusted to 1.5 × 10^6^ CFU/spot. The MIC was defined as the lowest concentration of SPF-B that permits microorganism growth after prior incubation at 37°C for 24 hr.

### 2.5. Synergistic Testing

The checkerboard method was used to identify the interactions between SPF-B and antibiotics [12]. The antimicrobial assays were performed with SPF-B in combination with AMP, OXI, GET, CIP, and NOR. Serial dilutions of SPF-B with these antibiotics were mixed in cation-supplemented MHB. The inocula were prepared from colonies that had been grown on MHA overnight. The final bacterial concentration after inoculation was 1.5 × 10^6^ CFU/spot. The MIC, determined after incubation at 37°C for 24 hr, was defined as the lowest concentration of drug, alone or in combination with other agents, that visibly inhibited the growth of bacteria. Each experiment was performed 3 times. The *in vitro* interaction between the drugs was quantified by determining the fractional inhibitory concentration (FIC). The FIC index (FICI) was calculated with the following formula:

(1) $$\begin{array}{c} \text{FIC}\;\text{index}\;={\text{FIC}}_{A}+{\text{FIC}}_{B}=\frac{\left[A\right]}{{\text{MIC}}_{A}}+\frac{\left[B\right]}{{\text{MIC}}_{B}}, \end{array}$$

where [*A*] is the concentration of drug *A* and MIC_*A*_ and FIC_*A*_ are the MIC and the FIC, respectively, of drug *A*, whereas [*B*], MIC_*B*_, and FIC_*B*_ are similarly defined for drug *B*. The FIC index thus obtained was interpreted as follows: values <0.5 denoted synergy; 0.5–0.75 partial synergy; 0.76–1 an additive effect; 1–4 no effect; and >4 antagonism. Finally, the different values of synergy between the 2 agents were calculated.

### 2.6. Time-Kill Assay

Time-kill curves were used to determine the synergy effects of the 2 antimicrobial agents on bacterial growth in 96-well plates at 5 different points of time (0, 4, 8, 16, and 24 hr) [12]. Bacterial cultures diluted with fresh MHB to approximately 1.5 × 10^6^ CFU/mL, and the diluted cultures were incubated at 37°C for 24 hr. Aliquots (0.1 mL) of the culture were taken at 0, 4, 8, 16, and 24 hr of incubation, and serial 10-fold dilutions were prepared in saline as needed. The numbers of viable cells were determined on a drug-free MHA plate after incubation for 24 hr. Colony counts were performed on plates, and 30–300 colonies were enumerated. The lower limit of sensitivity of the colony counts was 100 CFU/mL. The antimicrobial agents used were considered bactericidal at the lowest concentration that reduced the original inoculum by 3 log10 CFU/mL (99.9%) for each of the indicated times. However, they were designated bacteriostatic if the inoculum was reduced by only 0–3 log10 CFU/mL. To confirm the results, the time-kill assays for each experiment were performed at least thrice; the data are represented as mean data ± standard deviation. 

## 3. Results

### 3.1. The MICs of Sophoraflavanone B

Antimicrobial susceptibility tests of SPF-B against 7 strains of *S. aureus* were performed using the standard broth microdilution method. The MICs of SPF-B for each of the tested strains are presented in Table 2. The growth of *S. aureus* was inhibited in the range of concentrations from 15.6 to 31.25 *μ*g/mL SPF-B.

### 3.2. Synergistic Testing

The synergistic effects of SPF-B with various antibiotics were tested on MRSA strains by using a checkerboard dilution assay. The antibacterial effects of each separate SPF-B and the antibiotics and of SPF-B combined with the different antibiotics—AMP, OXI, GET, CIP, and NOR—are shown in Tables 3, 4, 5, 6, and 7. The antibacterial activity of SPF-B markedly reduced the MICs of these antibiotics against *S. aureus* strains. In combination with SPF-B, the MICs of AMP, OXI, GET, CIP, and NOR were reduced 2- to 16-fold, 2- to 32-fold, 8- to 32-fold, 2- to 32-fold, and 2- to 4-fold, respectively.

### 3.3. Time-Kill Curve Assay

**Figure 2:**
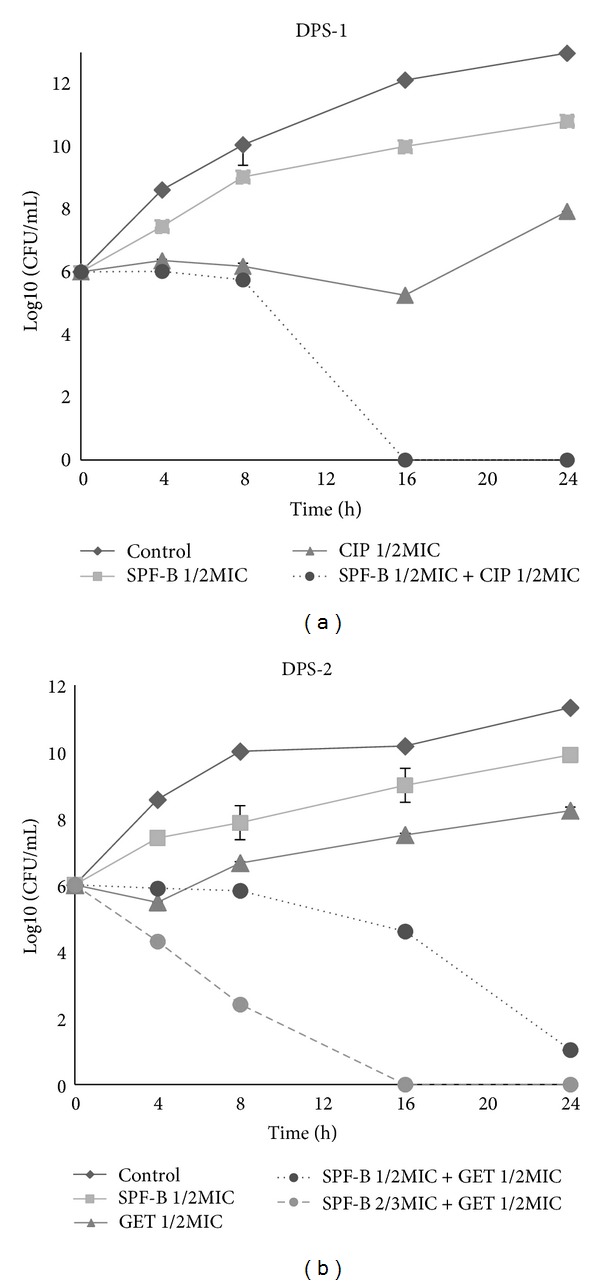
Time-kill curves of MRSA using SPF-B with CIP and GET.

The synergistic effects of SPF-B with selected antibiotics on MRSA were confirmed with a time-kill curve assay.  Figure 2 shows that, within a 24 hr incubation period, neither SPF-B alone nor an antibiotic alone induced cell death. However, when used together, the combination of SPF-B and an antibiotic caused rapid inhibition in a time-dependent process during an observation period of 24 hr. As shown in Figure 2, the combination of 1/2MIC SPF-B + 1/2MIC CIP completely inhibited the growth of MRSA (DPS-1) after 16 hr. In the presence of MRSA (DPS-2), the combination of 1/2MIC SPF-B + 1/2MIC GET reduced bacterial count by 5 log10 CFU/mL, and the drug concentration of 2/3MIC SPF-B + 1/2MIC GET completely inhibited the growth of MRSA (DPS-2) after 16 hr incubation. The enhanced effects of the combination of SPF-B + CIP and SPF-B + GET sharply reduced the number of CFUs.

## 4. Discussion

The emergence of multidrug-resistant (MDR) strains of *S. aureus *constitutes a serious problem worldwide. About fifty years ago methicillin-resistant *Staphylococcus aureus* (MRSA) was first discovered, and numerous research have been written about MRSA and constantly studied and analyzed by many medical science researcher. However, despite these efforts, MRSA have ceaselessly spread throughout the world and antibiotic resistance is increasing rapidly. Therefore, new therapeutic approaches into overcoming bacterial drug resistance is necessary, to develop alternative antibacterial drugs for the treatment of MRSA infections.

The goal of the present research was to investigate an antibiotic combination therapy of SPF-B against MRSA, in order, finally, to overcome the problem of multiple drug resistance. The first study, a susceptibility test, determined MICs of SPF-B against MRSA to be 15.6 to 31.25 *μ*g/mL. This experimental method showed clearly that SPF-B was a potent MRSA growth inhibitor (Table 2). We found that the use of various antibiotics—AMP, OXI, GET, CIP, and NOR—in combination with SPF-B caused an important reduction in the growth of MRSA (Tables 3–7). That combination therapy proceeds by different pathways according to the antibacterial agent used against pathogenic infections [13]. The most common combination strategy is to use drugs, each of which inhibits a different bacterial pathway. In the present study, we chose AMP and OXI as inhibitors of cell wall synthesis, GET as newer aminoglycoside, which disrupts the function of the prokaryotic ribosome [14], and CIP and NOR as inhibitors of nucleic acid metabolism and DNA synthesis of bacteria [15, 16].

Many studies have been published on the use of synergism to inactivate microorganisms, including MRSA, in purified plant-derived compounds, plant oils, and antibiotics; they have included scrutiny of the synergistic effects of SPF-B and antibiotics against MRSA. Brehm-Stecher and Johnson observed that the sesquiterpenoids nerolidol, farnesol, bisabolol, and apritone enhanced the antibacterial action of antibiotics against *S. aureus* and *E. coli *[17]. Aqil et al. and Kondo et al. demonstrated the synergistic effects against MRSA of ampicillin with ethanol extracts of 10 different Indian medicinal plants and of oxacillin with quinic acid gallates from *Caesalpinia spinosa *[18, 19]. Grande et al. found that antimicrobial activity significantly increased when enterocin AS-48 was used in combination with the phenolic compounds against *S. aureus* [20]. Liu et al. and Chung et al. observed the synergistic antibacterial activities of kaempferol glycosides with fluoroquinolones on MRSA and of pentacyclic triterpenoids combined with methicillin or vancomycin on *S. aureus* [21, 22]. Time-kill curve assays demonstrated that this synergistic activity of SPF-B with an antibiotic enhanced the inhibition rate against MRSA, with complete inhibition after 16 hr (Figure 2). This combination therapy suggests that it can increase susceptibility to antibacterial action and, as well, reduce antibiotic-inducible resistance of bacteria.

The focus of these findings is on developing novel anti-MRSA drugs to directly address the problem of MRSA, which is resistant to conventional multidrugs. The present study suggested that SPF-B has potent antibacterial and synergistic activity and that it can reduce the resistance that conventional drugs have acquired to MRSA. Furthermore, SPF-B has a distinct potential to become a promising alternative, natural anti-MRSA drug.

## Figures and Tables

**Table 1:** Determination of the *mec*A gene status of the *Staphylococcus aureus* strains used in this study.

*S*. *aureus *	Class	*mec*A gene	*β*-Lactamase activity	Antibiotic resistance
ATCC 33591	MRSA	+	+	AM, OX
ATCC 25923	MSSA	−	−	−

Clinical isolates				
DPS-1	MRSA	+	+	AM, OX
DPS-2	MRSA	+	−	AM, OX
DPS-3	MRSA	+	+	AM, OX
DPS-4	MRSA	+	−	AM, OX
DPS-5	MRSA	+	−	AM, OX

DPS: staphylococcal strain from the Department of Plastic Surgery, Wonkwang University Hospital; +: positive; −: negative; AM: ampicillin; OX: oxacillin.

**Table 2:** The MIC of SPF-B against 7 strains of *Staphylococcus aureus. *

*S*. *aureus *	MIC (*μ*g/mL)
	SPF-B
MSSA (ATCC 25923)	31.25
MRSA (ATCC 33591)	15.6
MRSA (DPS-1)	15.6
MRSA (DPS-2)	31.25
MRSA (DPS-3)	31.25
MRSA (DPS-4)	31.25
MRSA (DPS-5)	31.25

**Table 3:** Results of the combination of SPF-B + AMP against MRSA.

*S*. *aureus *	Agent	MIC (*μ*g/mL)		FIC	FICI	Outcome
		Alone	SPF-B + AMP			
ATCC 33591	SPF-B	15.6	3.9	0.25	0.75	Partial synergy
	AMP	500	250	0.5		
DPS-1	SPF-B	15.6	3.9	0.25	0.75	Partial synergy
	AMP	250	125	0.5		
DPS-2	SPF-B	31.25	3.9	0.12	0.62	Partial synergy
	AMP	125	62.5	0.5		
DPS-3	SPF-B	31.25	15.6	0.5	0.62	Partial synergy
	AMP	250	31.25	0.12		
DPS-4	SPF-B	31.25	15.6	0.5	0.53	Partial synergy
	AMP	62.5	1.95	0.03		
DPS-5	SPF-B	31.25	7.8	0.25	0.75	Partial synergy
	AMP	62.5	31.25	0.5		

**Table 4:** Results of the combination of SPF-B + OXI against MRSA.

*S*. *aureus *	Agent	MIC (*μ*g/mL)		FIC	FICI	Outcome
		Alone	SPF-B + OXI			
ATCC 33591	SPF-B	15.6	3.9	0.25	0.75	Partial synergy
	OXI	500	250	0.5		
DPS-1	SPF-B	15.6	3.9	0.25	0.31	Synergy
	OXI	500	31.25	0.06		
DPS-2	SPF-B	31.25	7.8	0.25	0.75	Partial synergy
	OXI	500	250	0.5		
DPS-3	SPF-B	31.25	15.6	0.5	0.62	Partial synergy
	OXI	500	62.5	0.12		
DPS-4	SPF-B	31.25	7.8	0.25	0.31	Synergy
	OXI	1,000	62.5	0.06		
DPS-5	SPF-B	31.25	7.8	0.25	0.75	Partial synergy
	OXI	500	250	0.5		

**Table 5:** Results of the combination of SPF-B + GET against MRSA.

*S*. *aureus *	Agent	MIC (*μ*g/mL)		FIC	FICI	Outcome
		Alone	SPF-B + GET			
ATCC 33591	SPF-B	15.6	3.9	0.25	0.31	Synergy
	GET	500	31.25	0.06		
DPS-1	SPF-B	15.6	3.9	0.25	0.31	Synergy
	GET	500	31.25	0.06		
DPS-2	SPF-B	31.25	3.9	0.125	0.25	Synergy
	GET	250	31.25	0.125		
DPS-3	SPF-B	31.25	7.8	0.25	0.31	Synergy
	GET	2,000	125	0.06		
DPS-4	SPF-B	31.25	3.9	0.125	0.25	Synergy
	GET	500	62.5	0.125		
DPS-5	SPF-B	31.25	7.8	0.25	0.375	Synergy
	GET	500	62.5	0.125		

**Table 6:** Results of the combination of SPF-B + CIP against MRSA.

*S*. *aureus *	Agent	MIC (*μ*g/mL)		FIC	FICI	Outcome
		Alone	SPF-B + CIP			
ATCC 33591	SPF-B	15.6	3.9	0.25	0.5	Synergy
	CIP	1,000	250	0.25		
DPS-1	SPF-B	15.6	3.9	0.25	0.31	Synergy
	CIP	500	62.5	0.06		
DPS-2	SPF-B	31.25	3.9	0.06	0.56	Partial synergy
	CIP	1,000	500	0.5		
DPS-3	SPF-B	31.25	15.6	0.25	0.5	Synergy
	CIP	500	125	0.25		
DPS-4	SPF-B	31.25	15.6	0.25	0.5	Synergy
	CIP	250	62.5	0.25		
DPS-5	SPF-B	31.25	7.8	0.25	0.75	Partial synergy
	CIP	250	125	0.5		

**Table 7:** Results of the combination of SPF-B + NOR against MRSA.

*S*. *aureus *	Agent	MIC (*μ*g/mL)		FIC	FICI	Outcome
		Alone	SPF-B + NOR			
ATCC 33591	SPF-B	15.6	3.9	0.25	0. 5	Synergy
	NOR	1,000	250	0.25		
DPS-1	SPF-B	15.6	3.9	0.25	0.75	Partial synergy
	NOR	500	125	0.5		
DPS-2	SPF-B	31.25	3.9	0.25	0.75	Partial synergy
	NOR	1,000	62.50	0.5		
DPS-3	SPF-B	31.25	15.6	0.25	0.5	Synergy
	NOR	1,000	31.25	0.25		
DPS-4	SPF-B	31.25	15.6	0.25	0.75	Partial synergy
	NOR	62.5	1.95	0.5		
DPS-5	SPF-B	31.25	7.8	0.5	0.75	Partial synergy
	NOR	500	31.25	0.25		
